# Super-resolution provided by the arbitrarily strong superlinearity of the blackbody radiation

**DOI:** 10.1038/s41467-019-13780-4

**Published:** 2019-12-17

**Authors:** Guillaume Graciani, François Amblard

**Affiliations:** 1 Center for Soft and Living Matter, Institute for Basic Science, Ulsan, South Korea; 2 Department of Physics and School of Life Sciences, Ulsan National Institute of Science and Technology, Ulsan, South Korea

**Keywords:** Applied physics, Optical physics, Super-resolution microscopy

## Abstract

Blackbody radiation is a fundamental phenomenon in nature, and its explanation by Planck marks a cornerstone in the history of Physics. In this theoretical work, we show that the spectral radiance given by Planck’s law is strongly superlinear with temperature, with an arbitrarily large local exponent for decreasing wavelengths. From that scaling analysis, we propose a new concept of super-resolved detection and imaging: if a focused beam of energy is scanned over an object that absorbs and linearly converts that energy into heat, a highly nonlinear thermal radiation response is generated, and its point spread function can be made arbitrarily smaller than the excitation beam focus. Based on a few practical scenarios, we propose to extend the notion of super-resolution beyond its current niche in microscopy to various kinds of excitation beams, a wide range of spatial scales, and a broader diversity of target objects.

## Abstract (web summary)

Here, the authors propose a new principle to achieve super-resolution for a wide class of targets. If an object is heated up by a beam, electromagnetic or acoustic, they show that the super-linearity of the induced thermal radiation leads to an arbitrarily high spatial compression factor relative to diffraction-limited beam profile.

## Introduction

Our capacity to resolve nearby objects with detection or imaging devices, such as optical microscopes, radars, and optical or radio telescopes, has been long recognized to be limited by diffraction. Since the pioneering work of Abbe in 1873^1^, the diffraction limit could not be overcome for more than a century. In the past 30 years, however, various concepts have been successfully proposed to bring the resolution of microscopy beyond the diffraction barrier^2–4^. One strategy is to use near-field excitation, with sub-micron optical apertures or tips that confine the excitation volume to sub-diffraction dimensions^5^. Near-field microscopies have successfully helped to resolve nanoscopic objects, but their field of application is rather limited because they do not defeat the diffraction limit per se in the far field.

For any kind of wave obeying the basic wave equation, the energy flow it transports cannot be focused in the far field to a focus smaller than the diffraction limit, and the resulting response of an object moving through that focus cannot be confined to a smaller spot unless one breaks the usually expected linear relationship between the local excitation intensity and the resulting response. However, if that linearity is broken by enhancing the response for high intensities and/or reducing it for low intensities, the response will necessarily be confined to a smaller volume. In the optical domain, such nonlinear responses require a saturable optical transition, which can be an intrinsic optical property of the target object, or the result of a spatially modulated saturation as implemented in STED (stimulated emission depletion) microscopy^6^. While such intrinsically nonlinear responses have been characterized for a range of photo-luminescent semiconductor objects, such as quantum dots^7^ or Si nanoparticles^8^, molecular fluorophores have also been successfully engineered for enhancing nonlinearities, leading to different methods of super-resolution microscopy^9^. However, beyond the context of photo-luminescence and super-resolved optical microscopy, the detection of any kind of object by its response to an electromagnetic excitation requires that a significant part of the excitation energy be scattered or converted into some sort of detectable radiative response. Non-radiative relaxation instead leads to heat production, which is useless in that context, except for some techniques such as photo-thermal imaging^10^.

The idea stands out here to consider that absorption-induced heating does not mean non-radiative relaxation, but necessarily produces instead a thermal radiation signal, which propagates in the far field. This excitation-induced thermal radiation could in principle be used for active thermal detection or imaging, as an attractive alternative for weakly scattering or weakly luminescent objects. This approach would best suit the case of strong absorbers with strong emissivity, idealized by the concept of blackbody objects with perfect absorption and maximal thermal radiation.

In the present report, we introduce a new idea that extends the concept of super-resolution beyond its original niche in fluorescence microscopy, to a broad range of potential applications in imaging and imageless detection. If a blackbody-like object is heated with a linear temperature rise by the energy it absorbs from the diffraction-limited focus of a beam, and regardless of the physical nature of that beam, its thermal response will be confined to a volume smaller than the focus. The theory we present shows indeed that the intrinsic superlinearity of the blackbody radiation spectrum theoretically leads to arbitrarily high spatial compression factors of the thermal response relative to the diffraction-limited excitation volume. Practically, the concept of super-resolved thermal detection is discussed for simple cases and in the context of recently observed heating probes and of the most advanced optical detectors.

## Results

### Nonlinear structure of the Planck radiation spectrum

While thermal radiation and the notion of a perfectly absorbing blackbody had been explored and partially understood since the pioneering work of Kirchhoff^11^, Planck proposed in 1901 a keystone heuristic argument, namely the quantification of light-matter interactions, that both initiated the development of quantum mechanics and solved the long-standing mystery called the ultraviolet (UV) catastrophe of blackbody radiation. The so-called Planck law of blackbody radiation^12^, which describes the spectrum of the thermal light emitted by a perfectly absorbing surface at thermodynamic equilibrium, can be expressed by the photonic spectral radiance, that is, the number of photons emitted per unit time, unit surface, unit solid angle, and per unit of wavelength (${\#}_{\mathrm{ph}}$ s^−1^ m^−2^ sr^−1^ μm^−1^), as:

1 $${\mathcal{S}}(T,\lambda )=2\times1{0}^{8}\frac{c}{{\lambda }^{4}}{\left({\exp }^{1{0}^{6}\frac{hc}{\lambda {k}_{\mathrm{B}}T}}-1\right)}^{-1},$$

where $T$ is the equilibrium temperature of the surface in Kelvins, $\lambda$ the wavelength in μm, $h$ and *k*_B_ the Planck and Boltzmann constants, and $c$ the speed of light. ${\mathcal{S}}(T,\lambda )$ reaches a spectral peak ${\mathcal{S}}_{\mathrm{max}}(T)$ that scales itself as $T^{4}$, for a wavelength ${\lambda}_{\mathrm{max}}(T)$ that scales as $T^{-1}$ according to Wien’s displacement law.

Let us now analyze the nonlinearities of the photonic spectral radiance ${\mathcal{S}}(T,\lambda )$ with respect to $T$ and $\lambda$. These nonlinearities can be characterized locally by two exponents $\nu_{\lambda }(T,\lambda )$ and ${\nu }_{T}(T,\lambda )$ defined by the local scaling ${\mathcal{S}}(T,\lambda )\propto {T}^{{\nu }_{T}}{\lambda }^{{\nu }_{\lambda }}$. In other words, for two dimensionless scalars $\varepsilon$ and $\eta$ with small enough values ($| \varepsilon | ,| \eta | \ll 1$), ${\mathcal{S}}((1+\varepsilon )T,(1+\eta )\lambda )\approx (1+{\nu }_{T}\varepsilon )(1+{\nu }_{\lambda }\eta ){\mathcal{S}}(T,\lambda )$. These two exponents, respectively, represent the ratios of the relative increase of ${\mathcal{S}}(T,\lambda )$ to the relative increases of $T$ and $\lambda$. Let us define $\alpha$ as a dimensionless scaling parameter. If the temperature is shifted from $T$ to $\alpha T$, the spectra at those two temperatures are related by the simple scaling relation:

2 $${\mathcal{S}}(\alpha T,\lambda )={\alpha }^{4}{\mathcal{S}}(T,\alpha \lambda ).$$

**Fig. 1:**
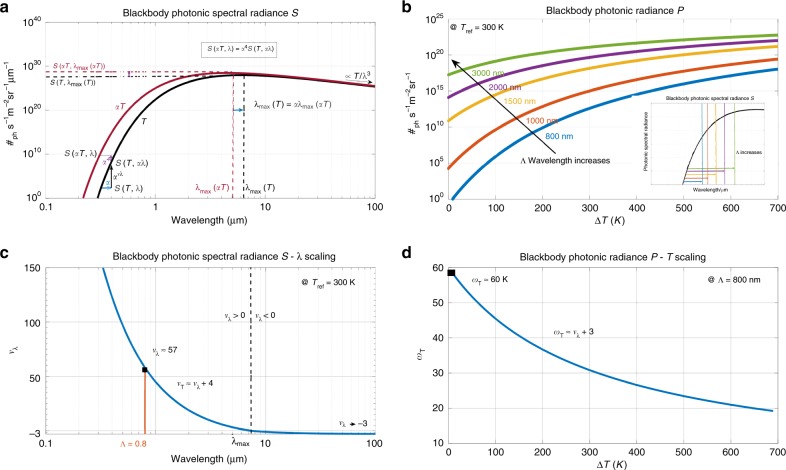
**Nonlinear structure of the Planck radiation spectrum.** **a** Blackbody photonic spectral radiance ${\mathcal{S}}(T,\lambda )$ in units of (${\#}_{\mathrm{ph}}$ s^−1^ m^−2^ sr^−1^ m^−1^), as a function of the wavelength for two temperatures $T$ (400 K) and $\alpha T$ (500 K, $\alpha =1.25$). The maximum emissions occur at ${\lambda }_{\mathrm{max}}(T)$ and ${\lambda }_{\mathrm{max}}(\alpha T)$, respectively, such that ${\lambda }_{\mathrm{max}}(T)=\alpha {\lambda }_{\mathrm{max}}(\alpha T)$. **b** blackbody photonic radiance (${\#}_{\mathrm{ph}}\,$ s^−1^ m^−2^ sr^−1^) as a function of the temperature excess $\Delta T$ above ${T}_{\mathrm{ref}}$ = 300 K, for different values of the upper boundary $\Lambda$ of the spectral integration domain. The blackbody photonic radiance ${\mathcal{P}}({T}_{\mathrm{ref}}+\Delta T,\Lambda )$ is obtained by integrating the photonic spectral radiance ${\mathcal{S}}({T}_{\mathrm{ref}}+\Delta T,\Lambda )$ over different spectral domains $[0,\Lambda ]$ as displayed on the lower right insert. **c** The function ${\nu }_{\lambda }(T,\lambda )$ describes the local nonlinearity exponent of ${\mathcal{S}}(T,\lambda )\propto {\lambda }^{{\nu }_{\lambda }}$. It is numerically computed for ${T}_{\mathrm{ref}}\,=\,300$ K. **d** The function ${\omega }_{T}(T,\Lambda )$ describes the local nonlinearity exponent ${\mathcal{P}}(T,\Lambda )\propto {T}^{{\omega }_{T}}$. It is numerically computed for $\Lambda$ = 800 nm.

This equation means that the log–log plot representation of ${\mathcal{S}}(\alpha T,\lambda )$ is simply obtained from ${\mathcal{S}}(T,\lambda )$ by two logarithmic translations $\lambda \to \alpha \lambda$ and ${\mathcal{S}}\to {\alpha }^{4}{\mathcal{S}}$ (Fig. 1 a). This scaling invariance of the radiance is well illustrated in the Supplementary note 2. When combined with the definition of the exponents, that is, ${\mathcal{S}}(T,\lambda )\propto {T}^{{\nu }_{T}}{\lambda }^{{\nu }_{\lambda }}$, one easily obtains ${\nu }_{T}(T,\lambda )=4+{\nu }_{\lambda }(T,\lambda )$. This is an important result, which means that thermal spectra all have the same structure of nonlinearities, that only depends on the ratio $\lambda /{\lambda }_{\mathrm{max}}(T)$. For $\lambda /{\lambda }_{\mathrm{max}}(T)\gg 1$, ${\mathcal{S}}\propto \,T{\lambda }^{-3}$, that is, ${\nu }_{T}=1$ and ${\nu }_{\lambda }=-3$. Qualitatively, for decreasing wavelengths, the exponent ${\nu }_{\lambda }$ increases from −3 to 0 at ${\lambda }_{\mathrm{max}}$, and further increases to infinity. The dependence in $T$ has a stronger superlinearity, since ${\nu }_{T}=4$ for ${\lambda }_{\mathrm{max}}$, and exhibits a faster divergence toward the UV catastrophe (Fig. 1 a). Quantitatively, ${\nu }_{\lambda }$ was numerically assessed from the logarithmic derivative $\frac{\partial {\mathrm{log}}({\mathcal{S}})}{\partial {\mathrm{log}}(\lambda )}$ (Fig. 1 c). For *T* = 300 K and λ = 800 nm, we found the scaling ${\mathcal{S}}\propto \,{\lambda }^{\approx 57}$. The practical meaning of exponents is the following. At 800 nm, the spectral radiance increases by 57% when the temperature increases by 1% above the ambient temperature, and it typically doubles for a 2 K temperature increase.

To make use of the superlinearity of the blackbody radiation with the temperature, photons should ideally be collected over a spectral region $0\le \lambda \le \Lambda$ with high nonlinearity exponents, that is, with $\Lambda \le {\lambda }_{\mathrm{max}}$. We therefore introduce the notion of partial photonic radiance $\mathcal{P}(T,\Lambda )$ defined by the spectral integration up to the boundary $\Lambda$:

3 $${\mathcal{P}}(T,\Lambda )=\mathop {\int}\limits_{0}^{\Lambda }{\mathcal{S}}(T,\lambda ){\rm{d}}\lambda,$$

which easily leads to a new scaling relation:

4 $${\mathcal{P}}(\alpha T,\Lambda )=\mathop {\int }\limits_{0}^{\Lambda }{\mathcal{S}}(\alpha T,\lambda ){\rm{d}}\lambda ={\alpha }^{3}{\mathcal{P}}(T,\alpha \Lambda ).$$

When $\Lambda \to \infty$, Eq. (4) indicates that the total number of thermal photons is superlinear in $T$ as it scales as ${T}^{3}$, which is in agreement with the ${T}^{4}$ scaling given by the well-known Stefan–Boltzmann law for the total energy. However, for any finite integration boundary $\Lambda$ on the UV side of the Planck spectrum, that is, $\Lambda \le {\lambda }_{\mathrm{max}}$, the increasing superlinearity of ${\mathcal{S}}$ for decreasing wavelengths leads to the fact that the spectral integral in Eq. (3) is largely dominated by the contribution at the upper boundary ${\mathcal{S}}(T,\Lambda )$. As a consequence, the superlinearity of the spectral integral is characterized by a new scaling relation, ${\mathcal{{P}}}(\alpha T,\Lambda )={\alpha }^{{\omega }_{T}}{\mathcal{{P}}}(T,\Lambda )$, in which the scaling exponent can be approximated as ${\omega }_{T}\approx 3+{\nu }_{\lambda }(T,\Lambda )$. Numerically, this nonlinearity exponent ${\omega }_{T}$ was assessed from the logarithmic derivative $\frac{\partial {\mathrm{log}}({\mathcal{P}})}{\partial {\mathrm{log}}(T)}$ (Fig. 1 d). For *T* = 300 K and $\Lambda =$ 800 nm, we found that ${\mathcal{{P}}}\propto \,{T}^{\approx 60}$, in agreement with ${\omega }_{T}\approx 3+{\nu }_{\lambda (T,\Lambda )}$.

### Compression of the point spread function

Let us now examine why and the circumstances under which the superlinearity described above can lead to super-resolved detection and imaging. If an extended object is locally heated up by a focused beam of energy characterized by a transverse intensity profile $I(x,y)$, the surface temperature will locally rise accordingly, but heat will be driven away from where it is initially created, by heat diffusion within the surface and perpendicular to it. However, if energy is delivered by short enough pulses, heat accumulation can be made faster than the relevant diffusion times, and the resulting thermal radiation can be captured from the focus before its spatial profile is broadened by diffusion. Pulsed illumination and/or fast beam scanning is therefore the preferred option for experimental realizations, and the following analysis will be performed under the assumption that heat does not diffuse away. In addition, we will assume that the spectral photonic radiance is determined by the local thermodynamic temperature of the surface and Planck’s law, despite the surface being far from equilibrium. This is a valid assumption for time-scales larger than the phonon equilibration time (1–10 ps). For the sake of simplicity, we make the assumption that the temperature locally rises proportionally to the intensity $I(x,y)$, that is, with no change of heat capacity and no phase transition. Also, the spectral emissivity will be considered to be invariant with temperature. Finally, a change of the target temperature may cause a variation of the spectral emissivity and thermal expansion. The emissivity comes as a wavelength-dependent pre-factor of the Planck spectrum, which possibly varies with temperature, although very smoothly. Emissivity was therefore assumed to be unity at all wavelengths. For most solid materials, expansion coefficients are typically $1{0}^{-5}-1{0}^{-6}$, and rarely exceed $1{0}^{-4}$. In the worst-case scenario, a 100 K increase would typically degrade the point spread function (psf) by a factor 1.01, and thermal expansion can be safely neglected.

Under the above assumptions, energy absorption, heat production, and temperature increase operate locally and linearly, and the notion of psf becomes meaningful for the following analysis. Let us consider a focused beam with a two-dimensional spatial intensity profile $I(x,y)={I}_{\mathrm{max}}\tilde{I}(x,y)$ such as a Gaussian or Airy shape, and an object smaller than the beam section. When located at position $(x,y)$, we assume that the object experiences uniform intensity $I(x,y)$ that produces a proportional temperature increase $\Delta T(x,y)=T(x,y)-{T}_{\mathrm{ref}}$, with ${T}_{\mathrm{ref}}\le T(x,y)\le {T}_{\mathrm{max}}$. Thermal detection over a given spectral domain $\lambda \le \Lambda$ can be described using the photonic radiance response, and we define the effective response as ${\mathcal{R}}(x,y)$ defined as the difference:

5 $${\mathcal{R}}(x,y)={\mathcal{P}}(T(x,y),\Lambda )-{\mathcal{P}}({T}_{\mathrm{ref}},\Lambda ).$$

The normalized spatial profile of that response, $\tilde{{\mathcal{R}}}(x,y)={\mathcal{R}}(x,y){{\mathcal{R}}}_{\mathrm{max}}^{-1}$, then appear as a function of the dimensionless excitation $\tilde{I}(x,y)$ parametrized by ${T}_{\mathrm{ref}}$, $\Delta {T}_{\mathrm{max}}$ and $\Lambda$ as:

6 $$\tilde{{\mathcal{R}}}(x,y)={{\mathcal{R}}}_{\mathrm{max}}^{-1}[{\mathcal{P}}({T}_{\mathrm{ref}}+\Delta {T}_{\mathrm{max}}\tilde{I}(x,y),\Lambda )-{\mathcal{P}}({T}_{\mathrm{ref}},\Lambda )]=\tilde{{\mathcal{R}}}(\tilde{I}(x,y)).$$

**Fig. 2:**
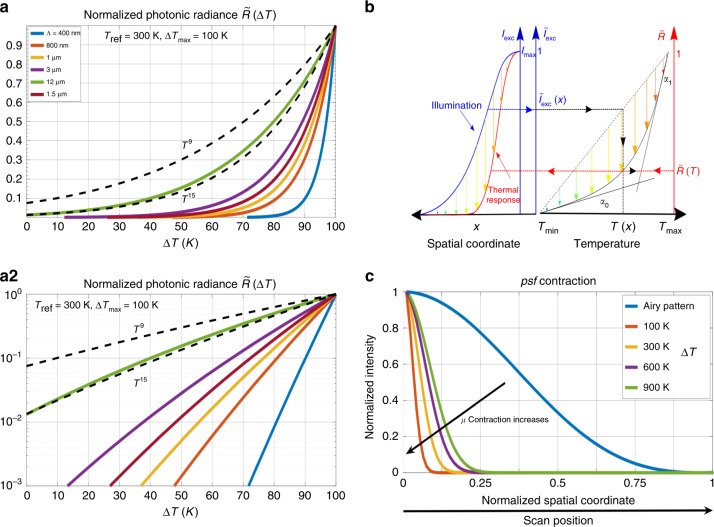
**Spatial compression of the excitation point spread function** (psf). **a** Normalized blackbody photonic radiance (in units of $\#_{\mathrm{ph}}$ s^−1^ m^−2^ sr^−1^) as a function of the temperature rise $\Delta T$ (0 K $\le \Delta {T} \le \Delta T_{\mathrm{max}}=$ 100 K), for different values of the maximum integration wavelengths $\Lambda$. **a1** is a linear plot, while **a2** is in semi-log scale. The photonic radiance is compared to two power laws ($T^{9}$ and $T^{15}$) of the temperature $T={T}_{\mathrm{ref}}+\Delta T$. **b** Spatial compression of an illumination psf (left, blue) resulting from the nonlinearity of Plancks law (right). The contracted profile of the effective thermal emission psf is represented on the left (red). The target temperature rises proportionally with the irradiance ${\tilde{\mathcal{I}}}_{\mathrm{exc}}$, and the thermal response $\tilde{\mathcal{R}}$ as a function of the temperature follows Plancks Law. **c** Example of the effect of thermal contraction on an Airy illumination psf (blue) for different values of $T_{\mathrm{max}}$—from left to right: 100, 300, 600, and 900 K—for a spectrum integrated up to $\Lambda =$ 1.5 μm.

This normalized dimensionless response $\tilde{\mathcal{R}}(\tilde{I}(x,y))$ is a complex non-algebraic function that exhibits a strong superlinearity (Fig. 2a1, a2) with apparent exponents that only depend on the parameters ${T}_{\mathrm{ref}}$, $\Delta {T}_{\mathrm{max}}$, and $\Lambda$. As a consequence, the spatial extension of $\tilde{\mathcal{R}}$ as a function of $(x,y)$ is contracted compared to $\tilde{I}(x,y)$, as schematically illustrated on Fig. 2b. The extent of psf contraction is assessed by the compression factor $\mu$ between these two profiles measured from their full width at $1/{e}^{2}$, which is computed numerically from Eq. (6), or by directly inverting that equation (see Supplementary Note 1). Numerical computations with $\Lambda$ = 1.5 μm indicate that the thermal radiation psf $\tilde {\mathcal{R}}(x,y)$ can be made 10-fold smaller than the excitation psf (Fig. 2d).

The above theoretical analysis shows that the superlinearity of the blackbody radiation response can be made arbitrarily high by moving the thermal detection to smaller wavelengths, and this leads to an arbitrarily high spatial compression factor between the cross-section of the excitation beam and the thermal radiation signal. Practically however, superlinearity is best achieved when $\Lambda \ll {\lambda }_{\mathrm{max}}({T}_{\mathrm{ref}})$, that is, when the spectral radiance becomes dramatically weak. Therefore, the photon budget must be assessed to optimize the trade-off between the detection sensitivity and the resolution.

### Photon budget and suggested conditions for super-resolved detection

**Fig. 3:**
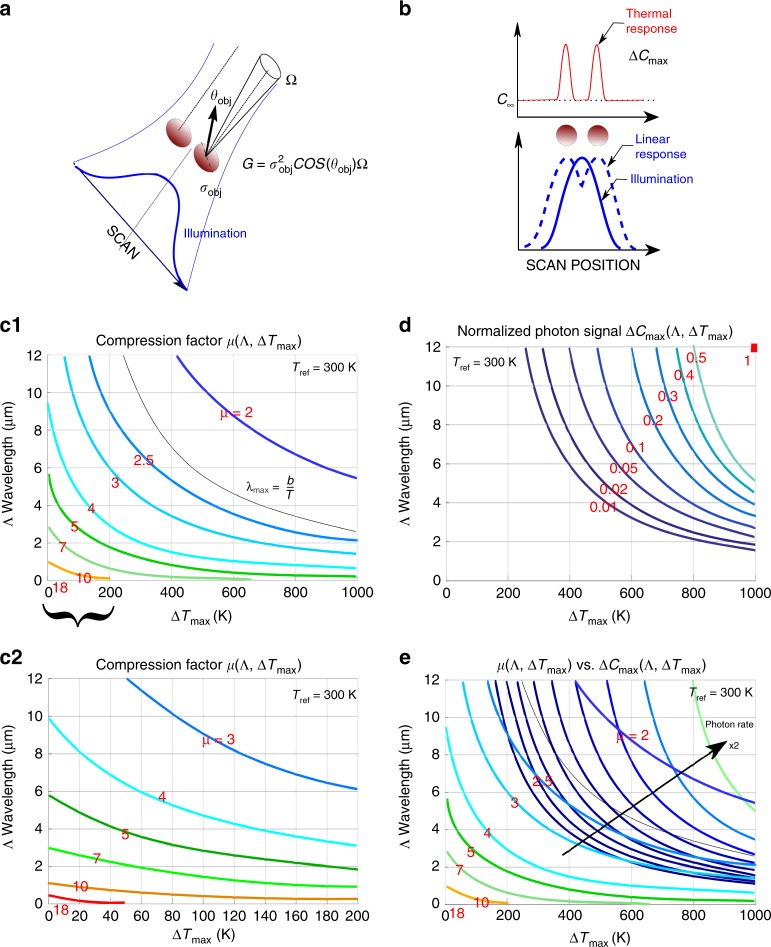
**Scanning illumination, thermal response, and compression factor.** **a** Two supposedly planar objects are illuminated by a focused beam with an excitation *psf* larger than their linear size ${\sigma }_{\mathrm{obj}}$ and the distance between them. Each object has an obliquity angle ${\theta }_{\mathrm{obj}}$ and supports an étendue $\mathcal{G}={\sigma }_{\mathrm{obj}}^{2}{\mathrm{cos}}({\theta }_{\mathrm{obj}})\Omega$. **b** The Planck thermal photon signal ${{\mathcal{C}}}_{\mathrm{max}}$ produced by the scanning illumination is compared to the linear response. ${{\mathcal{C}}}_{\mathrm{ref}}$ represents the background thermal photon signal. **c1**, **c2** The 2D plot of the psf compression factor $\mu$ as a function of the maximum heating $\Delta {T}_{\mathrm{max}}$ and the integration wavelength $\Lambda$. $\mu$ is computed for a Gaussian illumination psf as the ratio of the FWHM of the illumination psf and the thermal radiation psf, and shown for 0 K $\le \Delta {T}_{\mathrm{max}}\le$ 1000 K (**c1**) and 0 K $\le \Delta {T}_{\mathrm{max}}\le$ 200 K (**c2**). **d** Biparametric representation of the signal $\Delta {{\mathcal{C}}}_{\mathrm{max}}={{\mathcal{C}}}_{\mathrm{max}}-{{\mathcal{C}}}_{\mathrm{ref}}$ as a function of $\Delta {T}_{\mathrm{max}}$ and $\Lambda$. $\Delta {{\mathcal{C}}}_{\mathrm{max}}$ is obtained by integrating the photonic radiance $\mathcal{P}$ over the area of the thermal emission psf, and normalized to its value for $\Delta {T}_{\mathrm{max}}=$ 1000 K and $\Lambda =12$ μm (top-right corner). **e** Optimum of the trade-off of the photon budget and the resolution as a function of $\Delta {T}_{\mathrm{max}}$ and $\Lambda$, for ${T}_{\mathrm{ref}}=$300 K. Labeled and unlabeled lines represent constant values of $\mu$ and $\Delta {{\mathcal{C}}}_{\mathrm{max}}$, respectively. Each unlabeled line corresponds to a value of $\Delta {{\mathcal{C}}}_{\mathrm{max}}$ twice larger than the previous line.

For the sake of the present argument, we consider a beam scanning excitation scheme and a target object that contains details smaller than the beam cross-section. The thermal signal is not imaged, but is collected instead with a point detector, for each position $(x,y)$ of the target relative to the beam center (Fig. 3a). The signal is a number of photons per second ${\mathcal{C}}(x,y)$ given by:

7 $${\mathcal{C}}(x,y)={\mathcal{G}}\ \ {\mathcal{P}}({T}_{\mathrm{ref}}+\Delta {T}_{\mathrm{max}}\tilde{I}(x,y),\Lambda ),$$

where the étendue ${\mathcal{G}}$ of the optical system is proportional to the apparent cross-section of the object seen by the detector and the solid angle of detection. While the object is scanned, the signal of interest is collected as a temporal profile ${\mathcal{C}}(t)$, in which the target should produce a detectable variation from ${\mathcal{C}}_{\mathrm{ref}}$ due to the background temperature to ${\mathcal{C}}({T}_{\mathrm{max}})$ (Fig. 3b). The relevant signal is therefore the differential count rate ${\Delta} {\mathcal{C}}={\mathcal{C}}-{\mathcal{C}}_{\mathrm{ref}}={\mathcal{G}} \, {\mathcal{R}}$, and the number of signal photons is the integral $\int_{\mathrm{scan}}{\Delta} {\mathcal{C}}{\mathrm{d}}{t}$, which can be approximated by the product of the maximal rate $\Delta {{\mathcal{C}}}_{\mathrm{max}}$ by the typical time width ${\tau }_{\mathrm{emission}}$ of the peak emission. Meanwhile, the resolution will be limited by the psf compression factor $\mu$ introduced above. As expected, $\Delta {{\mathcal{C}}}_{\mathrm{max}}$ and $\mu$ vary in opposite ways with the temperature and the integration wavelength $\Lambda$ (Fig. 3c1, c2, d), and a trade-off must be arbitrated between the benefits of the spatial compression and the need of large enough photon rates (Fig. 3e).

Obviously, the integration time matters tremendously, as well as the optical étendue $\mathcal{G}$. Because the actual photon budget can only be meaningfully discussed if all parameters are considered, a few practical situations are examined at various scales (microscopic, macroscopic, mid-range, and long range), and for three different temperature amplitudes ($\Delta {T}_{\mathrm{max}}=$ 50, 100, and 150 K) above ambient temperature (${T}_{\mathrm{ref}}=$300 K) in Table 1. A compression factor $\mu$ between 5 and 10 is obtained, but the major role played by the optical étendue $\mathcal{G}$ is such that lower values of $\mathcal{G}$ must be compensated for by relatively larger detection times to achieve super-resolution. At any rate, the present analysis can serve the purpose of designing experimental tests of the new concept proposed here.

**Table 1:** Photon budget and psf thermal compression factor.

Excitation/detection parameters	$\mu$ Compression factor	Scale	Object size (${\sigma }_{\mathrm{obj}}$)	Optical system	Etendue ($\mathcal{G}$)	Planck signal ($\Delta {\mathcal{C}}_{\max }$)
Integration wavelength $\Delta =$ 1.5 μm ${T}_{\mathrm{ref}}=$ 300 K $\Delta T=$ 100 K	9.2	A: Microscopic	100 nm	NA = 1.2	10^−14^	1 ph s^−1^
		B: Macroscopic	5 μm	*D* = 4 cm *f* = 10 cm	10^−12^	10^2^ ph s^−1^
		C: Medium range	1 mrad diameter	10 cm^2^ receptor 45° obliquity	10^−8^	10^6^ ph s^−1^
		D: Long range	50 cm^2^ target	1 cm^2^ detector at *D*=500 m	10^−10^	10^4^ ph s^−1^
Integration wavelength $\Delta =$ 1 μm ${T}_{\mathrm{ref}}=$ 300 K $\Delta T=$ 150 K	7.4	A: Microscopic	100 nm	NA = 1.2	10^−14^	10^−3^ ph s^−1^
		B: Macroscopic	5 μm	*D* = 4 cm, *f* = 10 cm	10^−12^	10^−1^ ph s^−1^
		C: Medium range	1 mrad diameter	10 cm^2^ receptor	10^−8^	10^3^ ph s^−1^
				45° obliquity		
		D: Long range	50 cm^2^ target	1 cm^2^ detector at *D* = 500 m	10^−10^	10 ph s^−1^
Integration wavelength $\Delta =$ 1.5 μm ${T}_{\mathrm{ref}}=$ 300 K $\Delta T=$ 50 K	7.4	A: Microscopic	100 nm	NA = 1.2	10^−14^	10^−2^ ph s^−1^
		B: Macroscopic	5 μm	*D* = 4 cm, *f* = 10 cm	10^−12^	1 ph s^−1^
				10 cm^2^ receptor		
		C: Medium range	1 mrad diameter		10^−8^	10^4^ ph s^−1^
				45° obliquity		
				1 cm^2^ detector at *D* = 500 m		
		D: Long range	50 cm^2^ target		10^−10^	10^2^ ph s^−1^

For different spatial scales corresponding to different values of the optical étendue $\mathcal{G}$ (in units of m^2^sr as defined in Fig. 3a), we compute the compression ratio *μ* for a Gaussian illumination and the Planck signal ($\Delta{{\mathcal{C}}}_{\mathrm{max}}$ in ph s^−1^), for three temperature amplitudes ($\Delta {T}_{\mathrm{max}}$ = 50, 100, and 150 K) above $T_{\mathrm{ref}}$ = 300 K, and for two maximum integration wavelengths $\Lambda$ = 1.5 and 1 μm)

## Discussion

Historically, super-resolution has been almost exclusively developed for the field of optical microscopy by successfully taking advantage of nonlinearities offered by molecular spectroscopy. We propose here to break the diffraction limit by using the intrinsic superlinearity of the blackbody radiation, and demonstrate that it leads in theory to an arbitrarily large compression of the thermal radiation profile. Our analysis requires no assumption on the physical cause of the local temperature increase $\Delta T(x,y)$ nor the scale of the objects involved, opening up new applications far beyond thermal imaging and microscopy. Obviously, optically induced heating can be considered, but other mechanisms could be used, such as focused ultrasounds, for which the super-resolution argument holds. Regardless of the nature of the excitation beam, heat diffusion is likely to widen the psf of the thermal radiation, and pulse excitation schemes are therefore recommended (Supplementary Note 3).

The detailed analysis of the superlinearity is made here for the photonic spectral radiance ${\mathcal{S}}(T,\lambda )$ and not for the power spectral radiance ${\mathcal{S}}_{W}(T,\lambda )=\frac{hc}{\lambda }{\mathcal{S}}(T,\lambda )$. The reason is that quantum detector offer better performances than classical detectors for low light level applications, but a similar analysis can be made for the power spectral radiance, which leads to a slightly smaller but still quite significant superlinearity. We should also mention here that new quantum detectors, such as superconducting nanowire single-photon detectors, or HgCdTe avalanche photodiodes, could be instrumental to implement the ideas presented in this paper. HgCdTe avalanche photodiodes for instance provide indeed outstanding performances, with single-photon sensitivity, virtually no gain noise, and nano-second time resolution^13^ from the mid-infrared to the visible domain. Although we are not aware of probes designed so far to specifically serve as blackbody radiating probes, we anticipate that high emissivity biological pigments based on melanin, or recently proposed sub-micron probes that withstand high temperatures^14–16^, could be used to test our concept. In the context of these recent progresses on detectors and novel materials, the present work potentially broadens the scope of super-resolution beyond its historic microscopy niche, and should encourage its extension to a very broad spectrum of detection and imaging methods at all spatial scales and with a much broader diversity of objects.

## Methods

The mathematical derivation of this analytical work was done by hand while the numerical simulations were performed with basic Matlab routines.

## Supplementary information

Supplementary information

## Data Availability

The data that support the findings of this study are available from the corresponding author upon reasonable request.
