# Supplementary material for: Super-resolution provided by the arbitrarily strong superlinearity of the blackbody radiation
Source: Nat Commun. 2019 Dec 17;10:5761. doi: 10.1038/s41467-019-13780-4 (PMC6917796; doi:10.1038/s41467-019-13780-4)
Supplement: Supplementary file 1 — Supplementary information [file 41467_2019_13780_MOESM1_ESM.pdf]

Supplementary Information: Super-resolution provided by the arbitrarily strong superlinearity of the blackbody radiation

Graciani *et al.*

Supplementary note 1: Derivation of the *psf* compression factor μ

The purpose of this annex is to derive the formula we used to directly compute the compression factor μ induced by the superlinearity of the blackbody radiation spectrum.
As mentioned in the main text, the conditions for such a compression are as follow: a focused excitation beam with a 2D transverse spatial intensity profile $I(x, y) = I_{max}\tilde{I}(x, y)$, illuminates an object smaller than the beam waist. At each relative position (x, y), we assume that the object uniformly experiences the intensity $I(x, y)$ that produces a proportional temperature increase $\Delta T(x, y)$ relative to the background temperature T_{ref}. The object then emits a thermal signal determined by the photonic spectral radiance, i.e. the number of photons emitted per unit time, unit surface, unit solid angle, and per unit of wavelength, as:

$$\mathcal{S}(T, \lambda) = 2.10^8 \frac{c}{\lambda^4}\big(\exp^{10^6 \frac{hc}{\lambda k_B T}} - 1\big)^{-1} \#_{ph}\, s^{-1} m^{-2} sr^{-1} \mu m^{-1} \tag{1}$$

where T is the equilibrium temperature of the surface in Kelvins, λ the wavelength in microns, h and k_B the Planck and Boltzmann constants, and c the speed of light. The spectral integral of the spectral radiance over the wavelength detection window $[0, \Lambda]$ is called the photonic radianceand and reads:

$$\mathcal{P}(T, \Lambda) = \int_0^{\Lambda} \mathcal{S}(T, \lambda)\mathrm{d}\lambda \tag{2}$$

But T is a function of the position (x, y) of the target, and the spatial profile of the response $\mathcal{P}(T(x, y), \Lambda)$ can be compared to the illumination profile $I(x, y)$. We consider a Gaussian illumination and define the waist of these two profiles by the distance between their center the point where they reach $\frac{1}{e^2}$ of their maximum value. The compression factor μ is then defined as the ratio of the illumination waist to the thermal response waist. Other definitions of the waist using the $\frac{1}{e}$ or $\frac{1}{2}$ levels could be considered, with slightly different results.

By definition of the waist of the thermal response, it comes that:

$$\mathcal{P}(T_{thermal\, waist}, \Lambda) = \mathcal{P}(T_{ref}, \Lambda) + \frac{1}{e^2}(\mathcal{P}(T_{max}, \Lambda) - \mathcal{P}(T_{ref}, \Lambda)) \tag{3}$$

By definition of the compression μ and for the Gaussian illumination, we have:

$$T_{thermal\, waist} = T_{ref} + e^{-2/\mu^2}(T_{max} - T_{ref}) \tag{4}$$

Following the main text, let's consider $\alpha = \frac{T_{max}}{T_{ref}}$ and $\alpha_0 = \frac{T_{thermal\, waist}}{T_{ref}}$.
It comes that $\alpha_0 = 1 + e^{-2/\mu^2}(\alpha - 1)$, and we can then compute μ from the following equation:

$$\mathcal{P}(T_{thermal\, waist}, \Lambda) = \mathcal{P}(\alpha_0 T_{ref}, \Lambda) = \mathcal{P}(T_{ref}, \Lambda) + \frac{1}{e^2}(\mathcal{P}(\alpha T_{ref}, \Lambda) - \mathcal{P}(T_{ref}, \Lambda)) \tag{5}$$

Using the relation $\mathcal{P}(\alpha T, \Lambda) = \alpha^3 \mathcal{P}(T, \alpha\Lambda)$ (see equation (4) in main text) we obtain:

$$\alpha_0^3 \mathcal{P}(T_{ref}, \alpha_0\Lambda) = \mathcal{P}(T_{ref}, \Lambda) + \frac{1}{e^2}(\alpha^3 \mathcal{P}(T_{ref}, \alpha\Lambda) - \mathcal{P}(T_{ref}, \Lambda)) \tag{6}$$

For known values of T_{ref}, T_{max} and Λ, the second member of the latter equation is determined, and the equation can be numerically solved for α_0. The compression factor μ is then explicitly computed from equation (5) and reads :

$$\mu = \left[\frac{1}{2}\log\frac{\alpha - 1}{\alpha_0 - 1}\right]^{-1/2} \tag{7}$$

For large values of the compression ratio, $T_{thermal\, waist} \approx T_{max}$ and $\alpha_0 \approx \alpha$. In such circumstances, assuming $\mathcal{P}(T_{ref}, \alpha_0\Lambda) \approx \mathcal{P}(T_{ref}, \alpha\Lambda)$, equation (6) can be written an simplified as:

$$\alpha_0^3 \approx \left[\frac{\alpha^3}{e^2} + (1 - e^{-2})\frac{\mathcal{P}(T_{ref}, \Lambda)}{\mathcal{P}(T_{ref}, \alpha\Lambda)}\right] \tag{8}$$

For $T_{ref} = 300K$, $T_{max} = 400K$ and $\Lambda = 12\mu m$, we find that $\mu \approx 18$. The approximation $\alpha_0 \approx \alpha$ comes with a 0.15% error, while the approximation $\mathcal{P}(T_{ref}, \alpha_0\Lambda) \approx \mathcal{P}(T_{ref}, \alpha\Lambda)$ is better than 1%. In such circumstances, the compression factor can be explicitly computed from the following equation:

$$\mu = \sqrt{2}\left(\log(\alpha - 1) - \log\left(\left[\frac{\alpha^3}{e^2} + (1 - e^{-2})\frac{\mathcal{P}(T_{ref}, \Lambda)}{\mathcal{P}(T_{ref}, \alpha\Lambda)}\right]^{1/3} - 1\right)\right)^{-1/2} \tag{9}$$

And the quality of the approximation $\mathcal{P}(T_{ref}, \alpha_0\Lambda) \approx \mathcal{P}(T_{ref}, \alpha\Lambda)$ can be checked using equation 6.

--§--§--§--

Supplementary note 2: Scaling Invariance of the Planck's law

This annex provides a tilted double logarithmic representation that best shows the scaling invariance of the Planck's radiation spectrum.

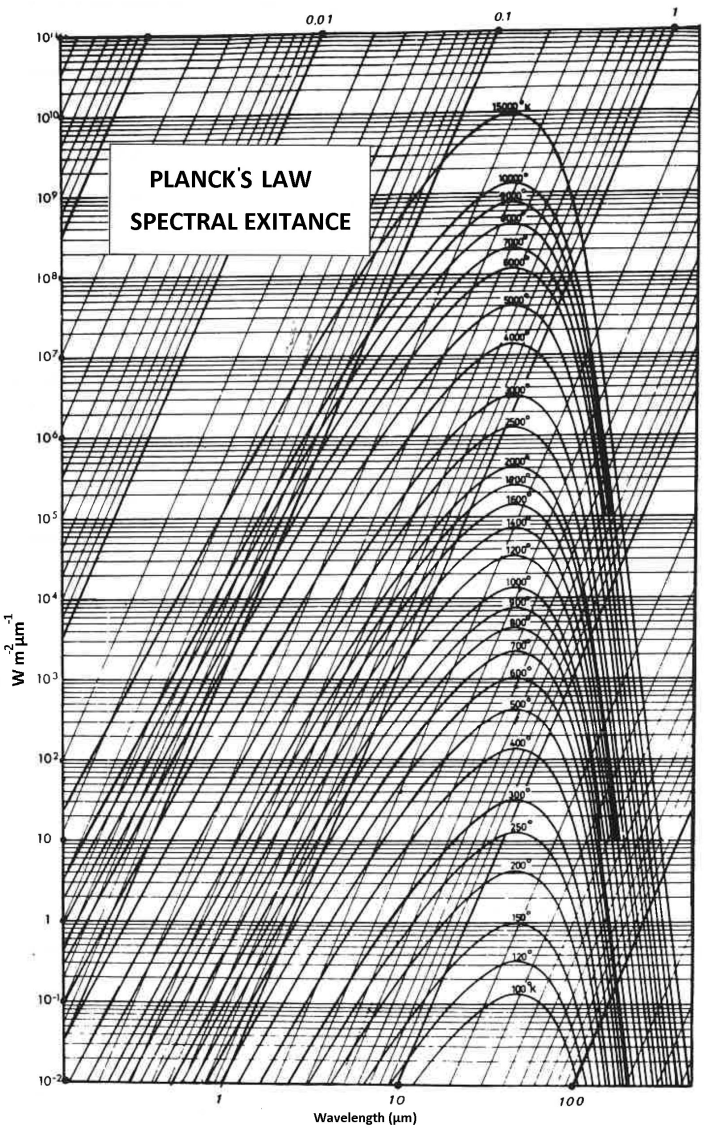

Supplementary figure 1: Planck's law - The spectral exitance, in units of $Wm^{-2}\mu m^{-1}$, corresponds to the flux emitted by the surface of a blackbody, per unit area and per wavelength. It is here represented for temperatures from 100K to 15,000K. This figure is from Gilbert Gaussorgues, *La Thermographie infrarouge : Principes, technologie, applications*, Editions Tec et doc, p20 (1999).

Supplementary note 3: Pulsed excitation scheme and temporal compression of the thermal radiation response

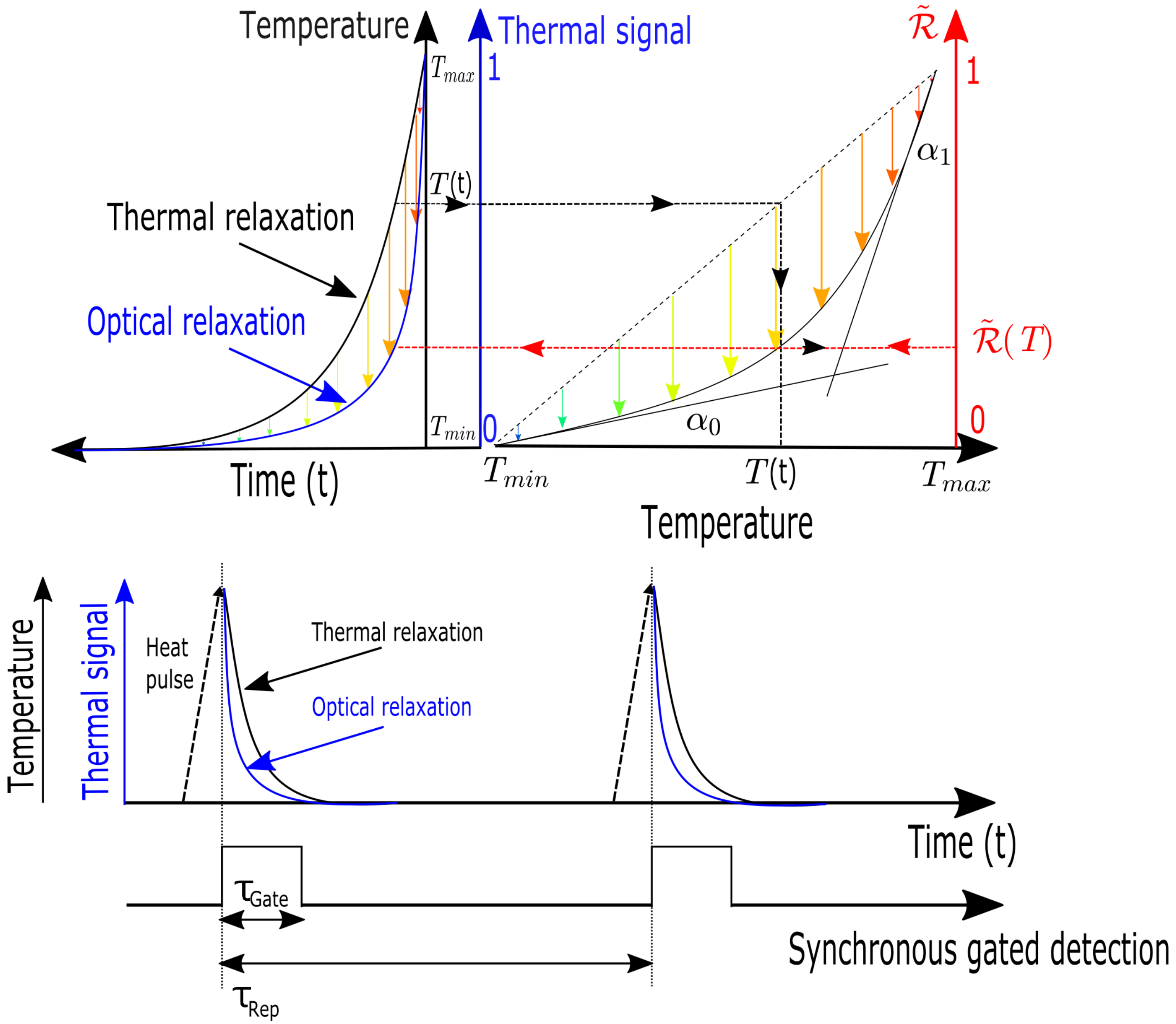

Supplementary figure 2: Pulsed excitation scheme and temporal compression of the thermal radiation response - **(top)** When the temperature of an object relaxes with time from a maximum T_{max} to an equilibrium temperature T_{min}, the superlinearity of the spectral radiance with temperature makes the former relax faster than the latter. This temporal compression has two practical consequences, when using a pulsed illumination. If the expected signal to noise ratio is an issue, gated acquisition will be the best approach, and we can use acquisition times that are shorter than heat relaxation times. On the contrary, if noise is not an issue, the strong superlinearity leads to a potentially very low noise levels, and the continous acquisition of the thermal radiation signal will be dominated by the radiation pulses. **(bottom)** Detection scheme of an induced thermal signal. An object undergoes a series of periodical heat pulses followed by thermal relaxations at τ_{Rep} intervals. The thermal radiation pulse can be detected by a synchronously gated detection of period τ_{Rep} and duration τ_{Gate}.

Supplementary note 4: Glossary

The symbol ˜ (tilde) accounts for the dimensionless normalized variables.

- c: Speed of light
- h: Planck constant
- k_B: Boltzmann constant

–

- I: Spatial intensity profile of the excitation beam
- T: Temperature; T_{ref}: Reference temperature before heating; ΔT: Difference between the temperature T and the reference temperature T_{ref}
- α: Relative increase in temperature, defined as the ratio of the new temperature to the original temperature
- λ: Wavelength
- Λ: Upper boundary of the spectral integration window
- $\mathcal{G}$: Optical étendue

–

- $\mathcal{S}$: Photonic spectral radiance ($\#_{ph}\, s^{-1} m^{-2} sr^{-1} \mu m^{-1}$)
- $\mathcal{P}$: Photonic radiance ($\#_{ph}\, s^{-1} m^{-2} sr^{-1}$), obtained from the spectral integral of the photonic spectral radiance ($\mathcal{S}$)
- $\mathcal{R}$: Spatial profile (x,y) of the thermal photonic radiance above background.
- $\mathcal{C}$: Effective thermal signal ($\#_{ph}\, s^{-1}$) obtained from the product of the Etendue and the photonic radiance; $\mathcal{C}_{ref}$: Thermal signal due to the background temperature ($\#_{ph}\, s^{-1}$); $\Delta\mathcal{C}$: Difference between the thermal signal $\mathcal{C}$ and its background level $\mathcal{C}_{ref}$ ($\#_{ph}\, s^{-1}$)
- ν_λ, ν_T: Local scaling exponents (as a function of T and λ) characterizing the nonlinearities of the photonic spectral radiance ($\mathcal{S}$) with respect to the wavelength λ and the temperature T
- ω_T: Exponent characterizing the nonlinearities of the photonic radiance ($\mathcal{P}$) with respect to the temperature T and defined as $\omega_T = 3 + \nu_\lambda$
- μ: Compression factor of the point spread function (psf)
- $\tau_{emission}$: Temporal width of the thermal emission pulses
